# Lipids modulate acetic acid and thiol final concentrations in wine during fermentation by *Saccharomyces cerevisiae* × *Saccharomyces kudriavzevii* hybrids

**DOI:** 10.1186/s13568-018-0657-5

**Published:** 2018-08-10

**Authors:** Amandine Deroite, Jean-Luc Legras, Peggy Rigou, Anne Ortiz-Julien, Sylvie Dequin

**Affiliations:** 1 Lallemand SAS, 31700 Blagnac, France; 2 SPO, Univ Montpellier, INRA, Montpellier SupAgro, Montpellier, France

**Keywords:** *Saccharomyces cerevisiae* × *Saccharomyces kudriavzevii* hybrids, Acetic acid, Thiols, Lipids, Box–Behnken experimental design, Wine fermentation

## Abstract

*Saccharomyces cerevisiae* × *Saccharomyces kudriavzevii* hybrids are typically used for white wine fermentation because of their cryotolerance. One group of these hybrids presents a unique ability to release thiol varietal aroma products as well as excessive amounts of acetic acid under specific conditions, which is detrimental for wine organoleptic quality. The aim of this work is to better assess the effects of lipids, sugar concentrations and temperature on the production of acetic acid and thiols during wine fermentation. To this end, we used a Box–Behnken experimental design and response surface modeling on the production of acetic acid and thiols in *S. cerevisiae* × *S. kudriavzevii* hybrids from the Eg8 family during fermentation of a synthetic must. We showed that these hybrids produced lower levels of acetic acid when the initial lipid concentration was increased, whereas they produced greater levels when the initial sugar concentration was high. Moreover, we found that lipids had a positive impact on the final concentrations of 4-methyl-4-mercaptopentan-2-one and 3-mercaptohexan-1-ol (3MH), giving box tree and citrus flavors, respectively. The increase of 3MH was concomitant with a decrease of 3-mercaptohexyl acetate (3MHA) characterized by a passion fruit aroma, indicating that lipid addition reduces the rate of 3MH acetylation into 3MHA. These results highlight the key role of lipid management in acetic acid metabolism and thiol release by *S. cerevisiae* × *S. kudriavzevii* hybrids and underline its technological interest in alcoholic fermentation to avoid the overproduction of volatile acidity while favoring the release of volatile thiols.

**Electronic supplementary material:**

The online version of this article (10.1186/s13568-018-0657-5) contains supplementary material, which is available to authorized users.

## Introduction

*Saccharomyces cerevisiae* wine strains form a cluster apart from *S. cerevisiae* strains from different origins, presenting genetic variations compared to *S. cerevisiae* from other ecological niches (Legras et al. 2007, 2018; Liti et al. 2009; Peter et al. 2018). This is a consequence of the historical and demographic events that the wine yeast populations have faced (Warringer et al. 2011). In addition to the population history, the constraints of the grape must composition and stresses imposed by wine fermentation conditions, such as high sugar concentrations, low pH, lipid and nitrogen deficiencies and high concentrations of ethanol, have led to specific adaptations, including interspecific hybridization (for a review, see Marsit and Dequin 2015). Many spontaneous interspecific hybridization events between *S. cerevisiae* and other *Saccharomyces* species have been detected among the isolates obtained from fermentations performed in cold climate areas. This is the case of *S. cerevisiae* × *Saccharomyces kudriavzevii* hybrids, which have been frequently isolated in low-temperature fermentations (for a review on *S. cerevisiae* × *S. kudriavzevii* hybrids, see Peris et al. 2018). Indeed, these hybrids combine the fermentative performances of *S. cerevisiae* and the cryophilic properties of *S. kudriavzevii,* which presents a lower optimal growth temperature (Belloch et al. 2008; Sampaio and Goncalves 2008; Salvadó et al. 2011). For example, a group of *S. cerevisiae* × *S. kudriavzevii* hybrids spread throughout Northern European vineyards, where grapes are frequently harvested in the autumn when temperatures are cooler (Erny et al. 2012). The Eg8 industrial strain, which belongs to this group, was originally isolated in 1979 from a vat fermenting at low temperature in Alsace (France) and was described as a triploid *S. cerevisiae* × *S. kudriavzevii* hybrid for the first time in 2012 (Erny et al. 2012). This strain and its variants, further called the Eg8 family, are mainly used in white wine fermentations because of their cryophilic characteristics and because of their ability to release a high amount of varietal thiols under oenological conditions (Murat et al. 2001). These thiols contain aromatic compounds that produce sought-after aromas characteristic of Sauvignon Blanc white wines but are also contained in many white grape varieties such as Gewurztraminer, Riesling or Petit and Gros Manseng grape juices (Dagan 2006; Roland et al. 2010). Each varietal thiol brings a particular aroma to the wine: 4-methyl-4-mercaptopentan-2-one (4MMP) is characteristic of box tree aroma, while 3-mercaptohexan-1-ol (3MH) and 3-mercaptohexyl acetate (3MHA) give fruity aromas, citrus and passion fruit flavors, respectively. 4MMP and 3MH are mainly liberated in the medium from odorless precursors present in the grape must (Cysteine-4MMP, Glutathione-4MMP, Cysteine-3MH, and Glutathione-3MH) owing to a yeast beta-lyase (Howell et al. 2005; Thibon et al. 2008; Holt et al. 2011; Roncoroni et al. 2011), and 3MH can be acetylated into 3MHA by Atf1p, an acetyltransferase produced by the yeast (Roland et al. 2011).

However, in addition to the desired ability to release thiols and their good ability to complete alcoholic fermentation, it has been reported that strains from the Eg8 family sometimes produce excessive concentration of acetic acid [> 0.7 g/L according to Ribéreau-Gayon et al. (2006)] under specific oenological conditions, leading to an unacceptable organoleptic quality of wine. Among the hybrids from the Eg8 family, two groups can be distinguished: the higher acetic acid producers which are ancestral strains and the lower acetic acid producers which have been obtained by UV mutagenesis from the first cluster, and selected for lower acetic acid production.

During alcoholic fermentation, *S. cerevisiae* produces acetic acid via the pyruvate dehydrogenase (PDH) bypass, which converts pyruvic acid to acetyl coenzyme A (acetyl-CoA) through three enzymatic steps catalyzed by pyruvate decarboxylase, acetaldehyde dehydrogenase (ACDH) and acetyl-CoA synthase (Holzer and Goedde 1957; Pronk et al. 1994; Remize et al. 2000). Acetaldehyde produced from pyruvic acid is converted to acetic acid mainly by the cytosolic enzymes Ald6p and Ald4p and the mitochondrial enzyme Ald5p, leading to NADPH regeneration (Remize et al. 2000; Saint-Prix et al. 2004).

It is already known that acetic acid production by *S. cerevisiae* during wine fermentation is impacted by several environmental factors, such as the initial sugar concentration (Erasmus and van Vuuren 2009; Monk and Cowley 1984), lipid content (Belviso et al. 2004; Landolfo et al. 2010; Ochando et al. 2016; Rollero et al. 2015; Thurston et al. 1981; Varela et al. 2012) and temperature (Beltran et al. 2008; Monk and Cowley 1984). However, the combined effects of these environmental factors on acetic acid production in yeast have never been studied, and the factors affecting the production of this acid in *S. cerevisiae* × *S. kudriavzevii* hybrids are unknown.

The objective of this work is to study the combined effects of three environmental parameters, sugar concentration, lipid content and temperature, on acetic acid production and thiol liberation by *S. cerevisiae* × *S. kudriavzevii* hybrids of the Eg8 family. A Box–Behnken experimental design was used to limit the number of experiments and to build a model that described the effects of these environmental factors and their interactions (Box and Behnken 1960). This model allows the illustration of the results through response surface, highlighting the optimal conditions to decrease acetic acid production and enhance thiol liberation during wine fermentation performed by a *S. cerevisiae* × *S. kudriavzevii* hybrid. Results obtained with the Box–Behnken experimental design were then validated in natural grape must using a complete experimental design.

## Materials and methods

### Yeast strains and media

The *S. cerevisiae* × *S. kudriavzevii* wine yeast strains used in this work are described in Table 1 and are natural hybrids from the group Eg8 described previously (Erny et al. 2012).

**Table 1:** *S. cerevisiae* × *S. kudriavzevii* hybrids used in the study

Name	Origin	Yeast collection
Eg8_V0	Low temperature (i.e., 15 °C) fermenting vat, Eguisheim (Alsace, France)	INRA Montpellier
Eg8_V2	Mutant strain of the Eg8_V0 strain obtained by UV mutagenesis, selected for its lower acetic acid production	Lallemand
Eg8_V3	Mutant strain of the Eg8_V0 strain obtained by UV mutagenesis, selected for its lower acetic acid production	INRA Montpellier
Eg8_V4	Alsace (France)	Lallemand

Strains were maintained as frozen stocks [YPD: yeast extract 1% weight/volume (w/v), peptone 1% w/v and glucose 2% w/v with glycerol 15% v/v] at − 80 °C or for short-term storage on YPD agar medium at + 4 °C. Fermentation flasks were inoculated with 10^6^ cells/mL from an overnight YPD culture.

### Fermentation media and conditions

Fermentations of the Box–Behnken experimental design were performed in synthetic medium (SM) with 200 mg/L yeast available nitrogen (YAN), which mimics a standard grape juice, following protocols described by Bely et al. (1990). The pH was adjusted to 3.3 with 10 M NaOH.

We used three sugar concentrations of 50% glucose and 50% fructose (170, 210 and 250 g/L) and three levels of lipids (0.0133, 0.0333 and 0.0533% v/v), which were made from a 15 g/L stock solution of phytosterols (*β*-sitosterol, Sigma Aldrich 85451, Saint-Louis, Missouri, États-Unis) in Tween80 (50%) and ethanol (50%). The final phytosterol concentrations were 2, 5 and 8 mg/L, with 0.0067, 0.0167 and 0.0267% v/v Tween80, respectively.

In the case of Eg8_V2, four thiol precursors (Nyseos, Montpellier, France) were added to SM medium to obtain the following final concentrations: 100 µg/L Cys-3MH; 500 µg/L Glu-3MH; 16 µg/L Cys-4MMP; and 3 µg/L Glu-4MMP. Nyseos (Montpellier, France) performed thiol quantification for fermentations in natural grape must.

Fermentations in grape must were performed in triplicate with Sauvignon Blanc must from Gers, France (2017), which contained 180 g/L sugar and 250 mg/L YAN and presented a turbidity of 140 nephelometric turbidity unit (NTU). This grape must was used in its natural state and subjected to decantation to achieve a turbidity of 20 NTU and chaptalization to achieve a sugar concentration of 240 g/L (50% fructose and 50% glucose). For some experiments, we tested three levels of lipids (0.0133, 0.0333 and 0.0533% v/v of the stock solution previously presented) or the addition of Tween80 alone to dissociate the effect of phytosterols from that of fatty acids on acetic acid production and thiol release.

Fermentations were performed in 330 mL flasks, containing 250 mL of medium continuously agitated by a magnetic stirrer (350 rpm). Anaerobiosis was obtained by bubbling argon for 20 min into the medium and was maintained through the use of fermentation locks. The tested temperatures were 16, 20 and 24 °C. Fermentations were monitored through the release of CO_2_, measuring the weight loss automatically every hour by robot-assistance (Box–Behnken experimental design in synthetic grape must) or manually twice a day (complete experimental design in natural grape must). When 80% of the initial sugar was consumed, fermentations were sampled for further analyses.

### Population counting and viability

Cell concentrations were determined with an electronic particle counter (Beckman Coulter ZB-2, Margency, France). To avoid aggregates, which may bias cell counts, samples were sonicated by an ultrasonic generator before counting (Branson Sonifier 250, Danbury, Connecticut).

Cell viability was determined by flow cytometry using a C6 cytometer (Accuri, BD Biosciences, San Jose, CA). Propidium iodide (PI) from Calbiochem (Sigma-Aldrich, Saint-Louis, Missouri, États-Unis) was added to the cell suspension (final concentration: 1 µg/mL). Since PI is a fluorescent nucleic acid stain that cannot penetrate intact cell membranes, fluorescence data for cells stained by PI allow determination of the percentage of intact and fragile cells among all cells (Tesnière et al. 2013).

### Determination of metabolite concentrations

Acetic acid production was determined using HPLC (HPLC 1290 Infinity, Agilent Technologies, Santa Clara, California, USA) equipped with a Phenomenex Rezex ROA column (Agilent Technologies, Santa Clara, California, USA) at 60 °C. This analysis also allowed for the determination of ethanol, glycerol, succinic acid, pyruvic acid, glucose and fructose concentrations. The column was resolved isocratically with 0.005 N H_2_SO_4_ at a flow rate of 0.6 mL/min. Acetic acid and pyruvic acid concentrations were determined using a UV meter at 210 nm, while the concentrations of all the other compounds were determined using a refractive index detector. Chromatograms were analyzed with the Agilent ChemStation software (Agilent Technologies, Santa Clara, California, USA). Analyses were performed in duplicate. When different sugar concentrations were studied, the metabolite production yields were determined instead of the concentration. This yield is expressed in g (or mg) of metabolite produced per g of sugar consumed.

### Thiol quantification

#### Sample storage

To limit thiol oxidation during storage, samples were protected from oxygen by the addition of 50 mg/L SO_2_. The suspensions were centrifuged for 10 min (3000 rpm) at 4 °C. The supernatants were stored at 4 °C in 4 mL glass vials, completely filled, and quickly analyzed (with a maximum storage of 8 days for 3MH and 3MHA and 3 days for 4MMP).

#### Standard solutions

Quantifications were performed by stable isotope dilution assay (SIDA) using deuterated thiols at a set concentration (4MMP_d10_ 0.1 µg/L, 3MH_d2_ 1 µg/L and 3MHA_d5_ 0.3 µg/L) and external calibration using natural thiols (Sigma-Aldrich, Saint-Louis, Missouri, États-Unis). The stock solutions were prepared in absolute ethanol.

#### 4MMP quantification by solid-phase microextraction (SPME)–gas chromatography–mass spectrometry (SPME–GC–MS/MS)

The 4MMP concentration in samples was quantified using a method published by Dagan et al. (2014), which was optimized for the use of triple quadrupole. The 4MMP was derivatized directly in the wine sample for 45 min at 55 °C using EDTA, l-cysteine and *O*-methylhydroxylamine hydrochloride and then extracted by SPME for 30 min at 55 °C on a DVB/CAR/PDMS fiber previously conditioned at 250 °C for 12 min. Finally, the compounds were desorbed into the GC inlet at 250 °C for 3 min. Derivatized compounds were separated, identified and quantified using a Trace Ultra gas chromatograph (GC) equipped with a Triplus Autosampler and coupled with a triple quadrupole mass spectrometer TSQ 8000 detector from ThermoScientific (Austin, Texas, USA). Analysis and data treatment were monitored using the Xcalibur software (ThermoScientific, Austin, Texas, USA).

The GC was equipped with a J&W DB-WAX (60 m × 0.25 mm × 0.25 µm) column (Agilent, Santa Clara, California, USA). The carrier gas was helium with a constant flow rate of 1.2 mL/min, and the injector temperature was set at 250 °C. Injection was performed in splitless mode for 3 min and then operated at a split of 1/20. The source and transfer line temperatures were set at 250 °C. Ionization was performed by positive electronic impact (EI) at 70 eV, with argon being used for the second fragmentation.

#### 3MH and 3MHA quantifications by nano-liquid chromatography–mass spectrometry (NanoLC-MS/MS)

The concentrations of 3MH and 3MHA were determined using the method published by Roland et al. (2016), with *N*-phenylmaleimide being added to 2 mL of wine sample for thiol derivatization. Derivatized thiols were extracted and purified using a Bond Elut Plexa cartridge from Agilent (Santa Clara, California, USA). After the samples were prepared, 4 µL of the recovered derivatized thiols in 0.1% formic acid-containing water/acetonitrile (98/2) were injected on an HPLC-polymeric chip ProtID (ZORBAX 300SB-C18, 40 nL, 5 µm and 75 µm * 43 µm, 5 µm). Compounds were chromatographically eluted using a nano-LC Agilent Series 1260 Infinity (Agilent, Santa Clara, California, USA) with a mobile phase of (A) water and (B) a mixture of ACN/TFE (90/10), both containing 0.1% (v/v) formic acid. The HPLC-Chip cube interface was connected to an Agilent 6460 triple quadrupole mass spectrometer (Agilent Technologies, Waldbronn, Germany). The Agilent Mass Hunter ChemStation software (version B 04.01) was used for data acquisition and processing.

### Statistical analysis

Statistical analyses were performed with R version 3.2.3 (R Development Core Team 2011).

#### Box–Behnken experimental design

We used the rsm library to implement the experimental design (Lenth 2009).

We used a Box–Behnken experimental design based on 16 fermentations, including 4 central points to evaluate the reproducibility in order to study the combined effects of sugar amount, lipid concentration and temperature on yeast metabolism during wine fermentation (Table 2). The impact of these factors on yeast central carbon metabolism was studied with a focus on acetic acid production, fermentation kinetics, cell population, cell viability and thiol liberation at the end of the fermentation.

**Table 2:** Box–Behnken experimental design

Experiments	Lipid content (% v/v)	Temperature (°C)	Sugar (g/L)
1	0.0133	16	210
2	0.0533	16	210
3	0.0133	24	210
4	0.0533	24	210
5	0.0133	20	170
6	0.0533	20	170
7	0.0133	20	250
8	0.0533	20	250
9	0.0333	16	170
10	0.0333	24	170
11	0.0333	16	250
12	0.0333	24	250
13^a^; 14^a^; 15^a^; 16^a^	0.0333	20	210

^**a**^Central points of the experimental design were replicated 4 times

Each studied variable had three coded factor levels: a low level, coded − 1; a high level, coded 1; and a mid-level, coded 0.

The effect of the independent variables on a resultant value Y (for example, the acetic acid production) was modeled by a polynomial response surface:

$$Y\, = \,\beta_{0} \, + \,\beta_{1} x_{1} \, + \beta_{2} x_{2} \, + \,\beta_{3} x_{3} \, + \,\beta_{12} x_{1} x_{2} \, + \,\beta_{13} x_{1} x_{3} \, + \,\beta_{23} x_{2} x_{3} \, + \,\beta_{11} x_{1}^{2} \, + \,\beta_{22} x_{2}^{2} \, + \,\beta_{33} x_{3}^{2} \, + \,\varepsilon$$

where x_1_, x_2_, and x_3_ are the coded values of the studied variables (temperature, lipid initial concentration and sugar initial concentration), β_0_ is the intercept term, β_i_ is the linear coefficient, β_ii_ is the quadratic coefficient, β_ij_ is the interaction coefficient and ε is the error term. This model was represented by response surfaces.

#### Mean comparisons

To check the mean equality of the results obtained with the complete experimental design in natural grape must, we performed Student–Newman and Keuls (SNK) statistical tests using the library agricolae.

## Results

The combined effects of sugar amount, lipid concentration and temperature on yeast metabolism during wine fermentation are shown in Table 3 (Additional file 1: Table S1).

**Table 3:**
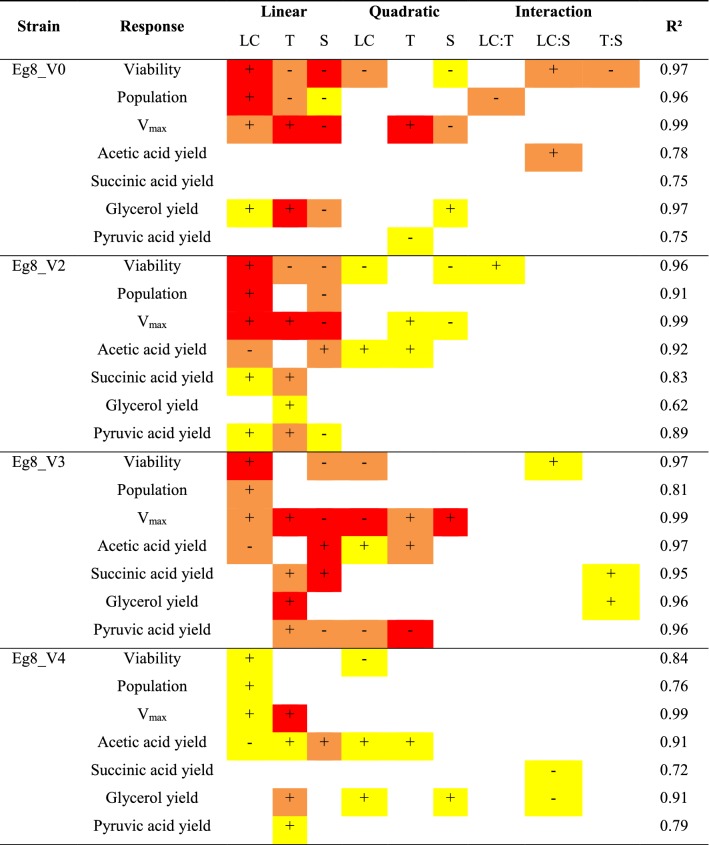
Combined effects of lipid concentration, temperature and sugar concentration on yeast growth, fermentation kinetics and carbon metabolism during wine fermentation

*LC* lipid content, *T* temperature, *S* sugar

Red: p < 0.001, orange: p < 0.01, yellow: p < 0.05, white: not-significant. (+): positive effect, (−): negative effect

### Effects of environmental conditions on the production of acetic acid and the main by-products of central carbon metabolism

#### Acetic acid

**Fig. 1:**
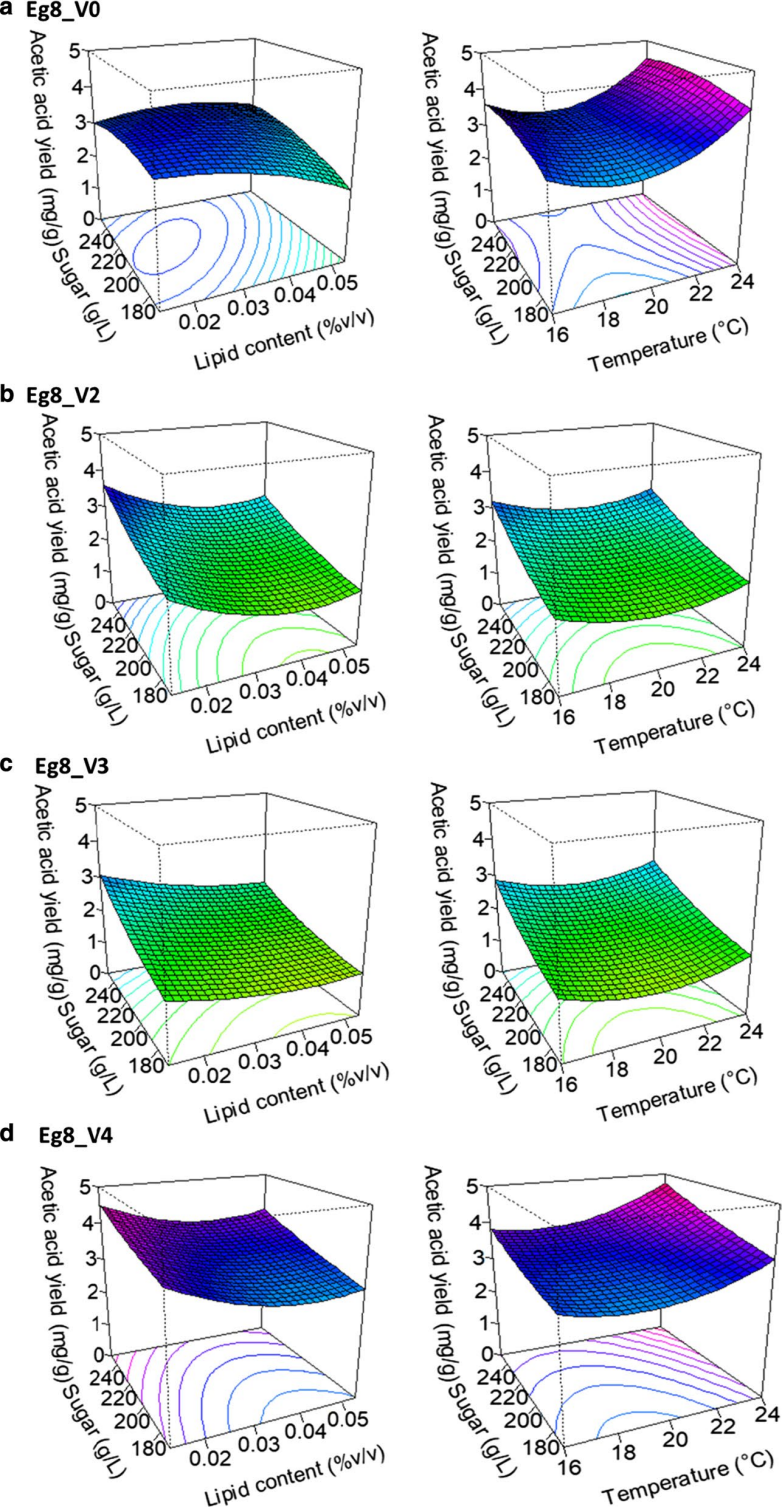
Evolution of acetic acid yield of *S. cerevisiae* × *S. kudriavzevii* hybrids Eg8_V0 (**a**), Eg8_V2 (**b**), Eg8_V3 (**c**) and Eg8_V4 (**d**) under different environmental conditions

The effects of environmental factors and conditions on the production of acetic acid by the 4 *S. cerevisiae* × *S. kudriavzevii* hybrids are shown in Fig. 1. These factors have different impacts on acetic acid production. Both the lipid content (Tween80 and phytosterols) and sugar concentration have great impacts on the acetic acid production of the four strains. For strains Eg8_V2, Eg8_V3 and Eg8_V4, acetic acid production increases with the initial sugar concentration in a linear manner and decreases with the lipid content of the must. In addition, the lipid content has a positive quadratic impact on the acetic acid production yield of these strains. In the case of Eg8_V0, the acetic acid production yield is also affected by the sugar concentration and lipid content but only when they interact; that is, the acetic acid production yield increases when high initial sugar concentration is associated with low initial lipid concentration.

In addition to the lipid content, temperature also affects the acetic acid production yield of the different strains, with the exception of Eg8_V0: the production increases with the temperature in a quadratic manner. According to the response surface (Fig. 1), the optimal temperature to reduce acetic acid yield in the tested conditions is approximately 20 °C.

As expected, the two low acetic acid producing mutant strains Eg8_V2 and Eg8_V3 have a lower acetic acid yield than Eg8_V0 and Eg8_V4, independent of the environmental conditions. Moreover, the acetic acid production of Eg8_V0 seems less affected by variations of the environmental conditions, suggesting that this strain regulates acetic acid metabolism differently compared to the other hybrids studied.

#### Glycerol, pyruvic acid, and succinic acid

Temperature has the main impact on metabolite production yields for the four hybrids. Indeed, glycerol production increases linearly with temperature for all strains. Temperature also affects pyruvic acid yield in the same manner, except in the case of Eg8_V0. Moreover, regarding pyruvic acid production by Eg8_V0 and Eg8_V3, different response surface profiles are observed compared to the other strains due to temperature having a negative quadratic impact (Additional file 1: Fig. S1).

The two low acetic acid producing mutant strains, Eg8_V2 and Eg8_V3, share similar responses to environmental factors. Temperature and sugar have a positive effect on glycerol yield as well as succinic acid production. Moreover, these two strains experience the same positive, linear effects of temperature and sugar concentration on succinic acid yield.

### Effects of environmental conditions on yeast growth, viability and fermentation rate

According to Table 3, the addition of lipids has a positive impact on the fermentations performed by these hybrids. This is illustrated by an increase in cell population and viability at the end of the fermentation and a higher maximum fermentation rate (V_max_). Moreover, the lipid content has a negative quadratic effect on the cell viability.

Conversely, the viability and fermentation rate (V_max_) decrease when the initial concentration of sugar increases for all the strains except for Eg8_V4. Sugar concentration also impacts the populations of Eg8_V0 and Eg8_V2. Eg8_V0 and Eg8_V2 behave similarly in regards to the quadratic effect of the sugar concentration, which negatively impacts their viability and V_max_. Finally, the viability of Eg8_V0 and Eg8_V2 is affected by a positive interaction between the lipid content and sugar concentration.

As expected, increasing the temperature to 24 °C strongly improves the fermentation rate (V_max_) for all the hybrids, even if they are cryophilic. This result is clearly illustrated by the response surface presented in Additional file 1: Fig. S2.

### Effects of environmental conditions on thiol liberation by Eg8_V2

Since the storage and quantification of thiols present significant challenges, we decided to perform in depth analysis of one single strain. The strain Eg8_V2, which produces low acetic acid levels, a desirable property for winemaking, was selected for this study.

**Fig. 2:**
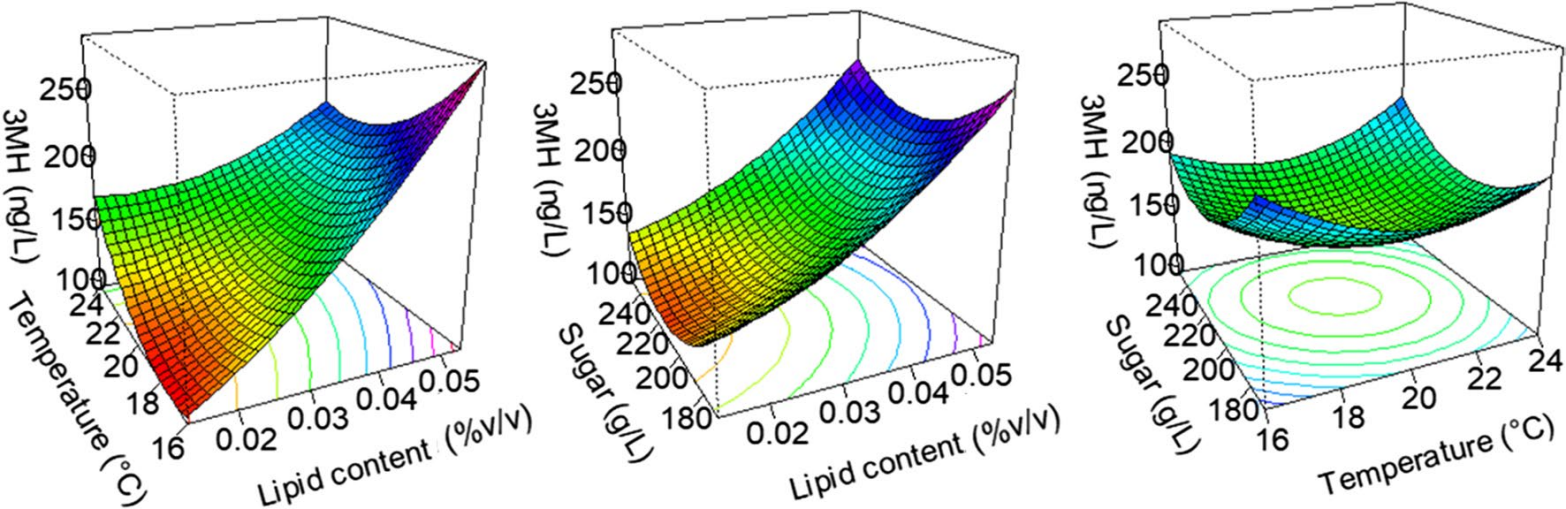
Effect of lipid content, sugar concentration and temperature on the liberation of 3MH by Eg8_V2

In synthetic media, we found a positive linear effect of the lipid content on 3MH liberation (Fig. 2). As thiols are volatile compounds present at low concentrations in wine and are difficult to store and quantify, we decided to confirm this effect and further explore the effects of other environmental factors on thiol release using a complete experimental design with fermentations in triplicate for all conditions. Moreover, all thiol precursors have not yet been discovered. Subileau et al. (2008) demonstrated that Cys-3MH was only responsible for 3–7% of the total 3MH in wine. Among these uncharacterized precursors, glutathionylated precursors are very likely to have a significant role. Indeed, Subileau et al. (2008) compared the Cys-3MH conversion yields obtained by Howell et al. (2004), Dubourdieu et al. (2006) and Masneuf Pomerede et al. (2006) and showed that the Cys-3MH conversion yield is higher in grape musts (up to 10%) than in synthetic media (below 1%). Therefore, our results obtained in a synthetic media may not mimic the reality. This prompted us to perform a complementary study in a natural grape must in order to take into account the full set of thiol precursors present in this must.

### Experimental validation of the effect of the lipid content of grape must on thiol release and acetic acid production

**Fig. 3:**
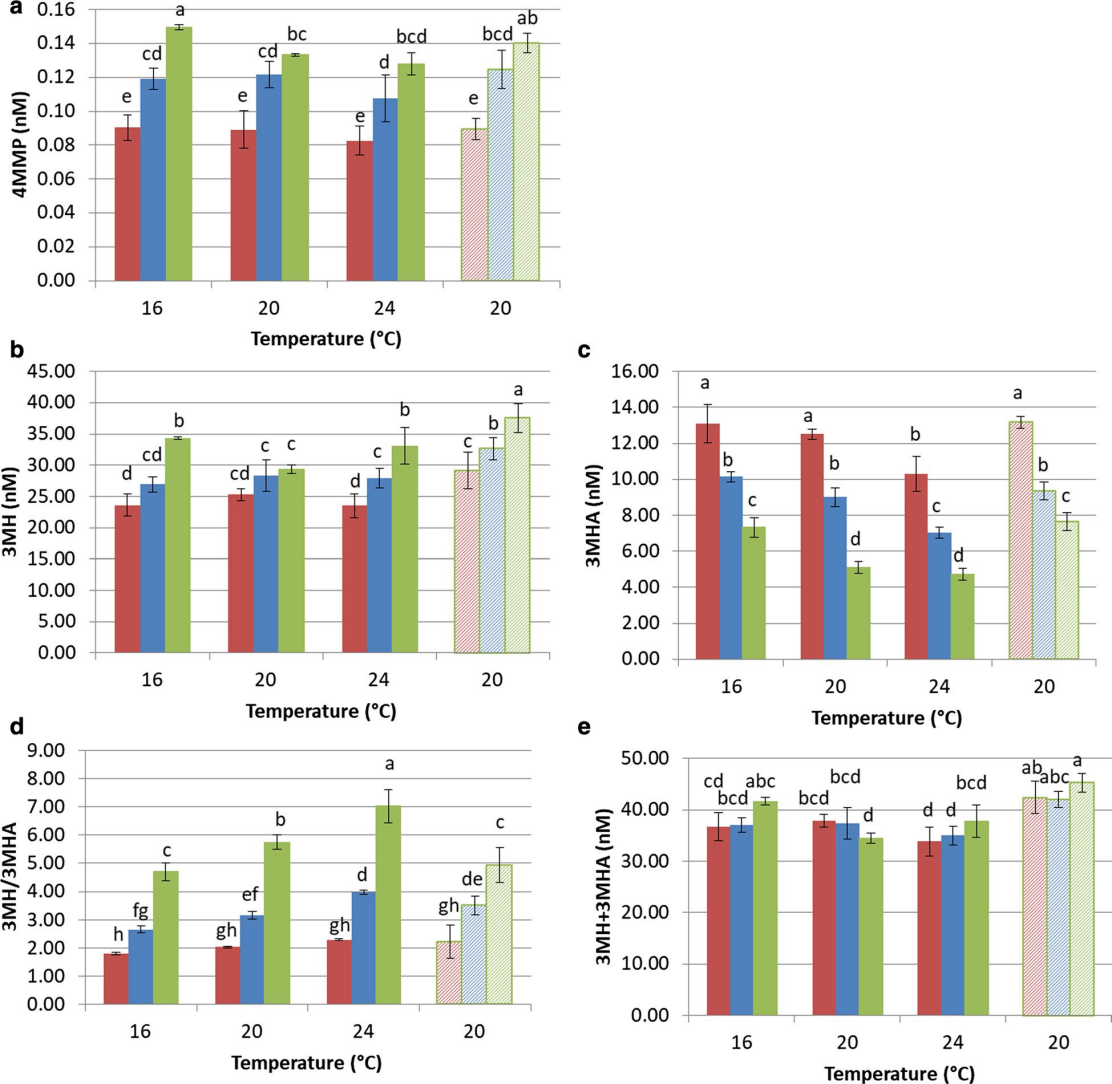
Impact of the initial concentration and nature of the lipid content and of the temperature on the production of 4MMP (**a**), 3MH (**b**) and 3MHA (**c**) and on the 3MH/3MHA ratio (**d**) and 3MH + 3MHA sum (**e**) during alcoholic fermentation by Eg8_V2 in Sauvignon Blanc du Gers 2017. Lipid content: red = 0.0133% v/v; blue = 0.0333% v/v; green = 0.0533% v/v; filled bar = phytosterols + Tween80; hatched bar = Tween80 only; letters above bars = groups generated with SNK test

**Fig. 4:**
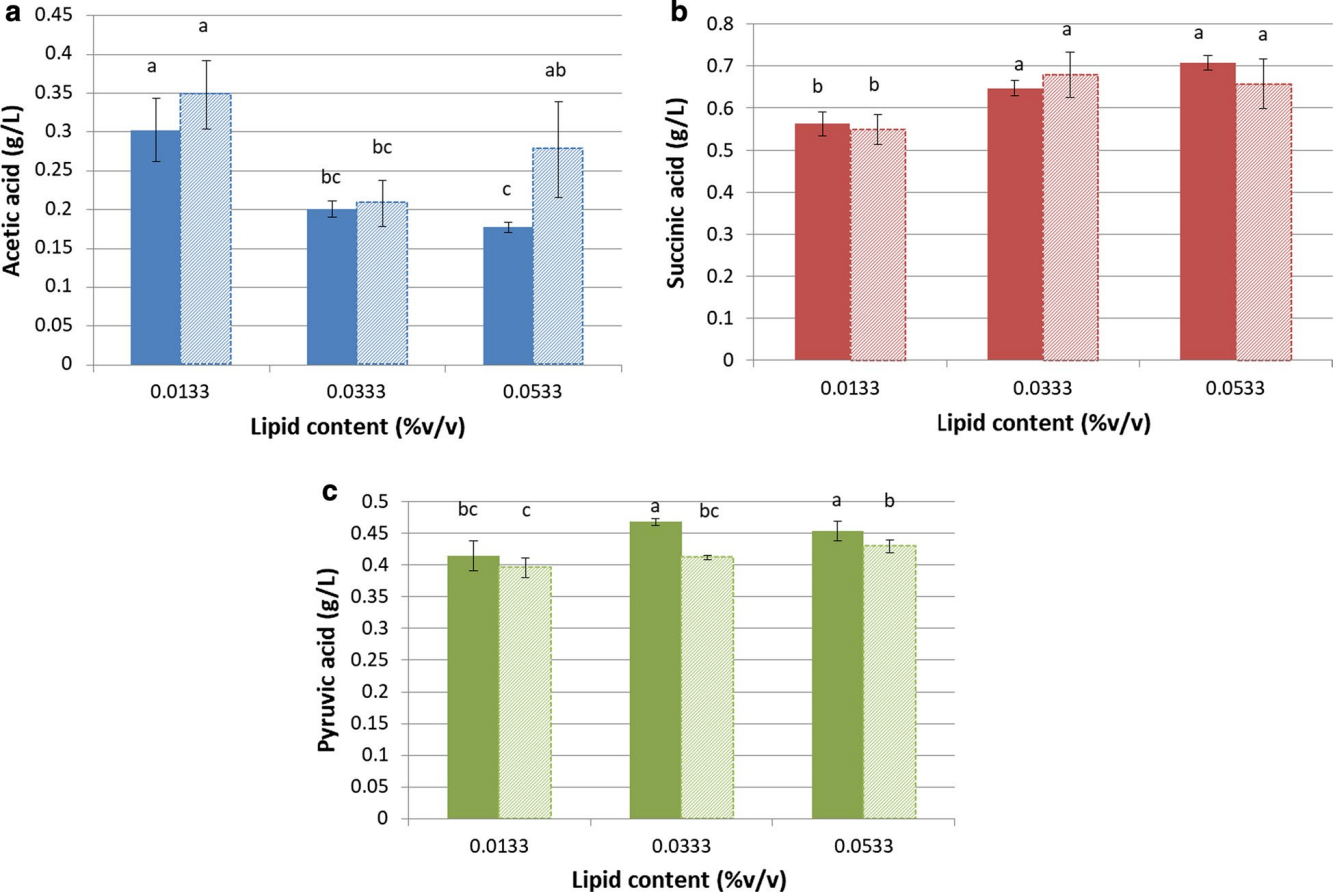
Impact of the initial concentration and nature of the lipid content on the production of acetic acid (**a**), succinic acid (**b**) and pyruvic acid (**c**) during alcoholic fermentation by Eg8_V2 at 20 °C in Sauvignon Blanc du Gers 2017 clarified until 20 NTU and chaptalized at 240 g/L sugar. Filled bar = phytosterols + Tween80; hatched bar = Tween80 only; letters above bars = groups generated with SNK test

We performed our complementary study using a Sauvignon Blanc grape must. As Twen80 contains fatty acids, the lipid effect observed in synthetic must can be due to phytosterols, fatty acids or both. To dissociate these effects, we tested the effect of Tween80 separately from that of phytosterols. We also tested the effects of temperature on the thiols released from grape precursors under these conditions. At the same time, we wanted to confirm the effects of these factors on acetic acid production. The results are presented in Figs. 3 and 4 (Additional file 1: Tables S2 and S3).

#### Impact of the lipid content and fermentation temperature on thiol release in natural grape must

Figure 3a, b clearly show that an increase in the lipid content of grape must, provided as either Tween80 + phytosterols or Tween80 alone, result in an increase in the amount of 3MH and 4MMP released by Eg8_V2 during alcoholic fermentation. Conversely, 3MHA is negatively impacted by the initial lipid concentration (Fig. 3c). As a result, the 3MH/3MHA ratio is multiplied by 3 between the modalities containing the lowest and highest lipid contents, independent of the fermentation temperature (Fig. 3d). We can hypothesize that lipid addition leads to a decrease in the acetylation of 3MH into 3MHA since the sum of the 3MH and 3MHA does not vary with the lipid concentration (Fig. 3e).

The release of 3MH and 4MMP is not affected by the fermentation temperature. In contrast, the 3MHA concentration decreases when temperature increases. Depending on the lipid content, the concentration of 3MHA decreases by 20–35% when the temperature increases from 16 to 24 °C (Fig. 3c). The 3MH/3MHA ratio increases with temperature (Fig. 3d).

#### Impact of the lipid content and fermentation temperature on acetic acid production in natural grape must

The effects of lipids on acetic acid production was studied in the grape must used for the evaluation of thiols and in that same grape must after clarification and enrichment to reach a sugar content of 240 g/L. Small differences in acetic acid concentration (between 0.11 and 0.17 g/L) were found in the untreated grape must (Additional file 1: Table S2), very likely because of its high turbidity (140 NTU). In contrast, a marked effect of lipids was observed in the clarified grape must; the acetic acid production decreased with higher initial concentrations of lipids. This effect was also observed when the lipid source was Tween80 alone. The impact was stronger when lipids were added in amounts between 0.0133 and 0.0333% v/v: acetic acid production dropped from 0.3 to 0.2 g/L (Fig. 4a and Additional file 1: Table S3). Moreover, this acetic acid production decrease accompanies an increase in succinic and pyruvic acids (Fig. 4b, c).

## Discussion

Eg8 is a *S. cerevisiae* × *S. kudriavzevii* natural hybrid mainly used in white wine fermentation because of its cryophilic character and its strong ability to release varietal thiols. However, Eg8 has been described as a high acetic acid producer. The aim of this work was to identify environmental conditions that could minimize acetic acid production while maintaining a high thiol concentration at the end of the fermentation. We focused our study on the impact of the initial sugar and lipid concentrations and temperature. To explore the effects of these three factors, we used a Box–Behnken experimental design, which enabled us to build a model describing the effect of each factor and their interactions.

We showed that acetic acid production by the hybrids decreases when the medium is supplemented with lipids, in both a synthetic must and a highly clarified grape must. A negative effect of lipid addition on the acetic acid production by *S. cerevisiae* had already been reported in several studies (Belviso et al. 2004; Delfini et al. 1992; Landolfo et al. 2010; Ochando et al. 2016; Rollero et al. 2015; Thurston et al. 1981; Varela et al. 2012). The main hypothesis to explain this relation between lipids and acetic acid production is that in anaerobiosis, when yeast is confronted with a deprivation of lipids in the medium, it synthetizes its own lipids from cytosolic acetyl-CoA (Parks and Adams 1978). Since the PDH bypass is the main metabolic pathway leading to cytosolic acetyl-CoA synthesis (Flikweert et al. 1996; Van Der Berg and Steensma 1995), it could be hypothesized that the lack of fatty acids and sterols in the medium leads to an increased pyruvic acid flux through the PDH bypass in order to produce more cytosolic acetyl-CoA. Since acetic acid is an intermediate of this pathway, its concentration increases when yeast faces a lipid deficiency in anaerobiosis. We also found that the initial lipid concentration influences the fermentation rate, yeast population and viability in a positive way for all the hybrids tested, as described previously for *S. cerevisiae* (Casalta et al. 2013; Houtman and Du Plessis 1981; Luparia et al. 2004; Ochando et al. 2016). Since clarification leads to a loss of sterols and fatty acids from the grape must (Cocito and Delfini 1997), this step of the winemaking process has to be controlled to avoid stuck fermentation and excessive production of acetic acid (Houtman and Du Plessis 1981; Delfini and Costa 1993).

In the case of Eg8_V2, the initial lipid concentration decrease also leads to a decrease in pyruvic acid and succinic acid concentrations in both the synthetic and natural grape musts. During wine fermentation, Krebs cycle enzymes are subjected to catabolite repression due to the high glucose concentration (Gancedo 2008), and the Krebs cycle functions as two branches with succinic acid as a final product (Gombert et al. 2001; Camarasa et al. 2003). The decrease in succinic acid concentration, coupled with higher acetic acid production, is consistent with a redistribution of the metabolic flux of central carbon metabolism from the Krebs cycle to the PDH bypass under conditions of lipid limitation. This supports the hypothesis that yeast use the cytosolic acetyl-CoA produced through the PDH bypass to produce their own fatty acids in order to counter lipid deficiencies.

Acetic acid production of Eg8_V2, Eg8_V3, and Eg8_V4 increases in a linear manner with the initial sugar concentration. This confirms results previously obtained for 8 *S. cerevisiae* strains in a natural grape must by Ferreira et al. (2006) and is very likely associated with a higher expression of *ALD4* and *ALD6* (Erasmus et al. 2003; Saint-Prix et al. 2004). Indeed, these two genes from the ACDH family are involved in the conversion of acetaldehyde into acetic acid in the PDH bypass (Remize et al. 2000). This might be related to osmotic stress impacting the pentose phosphate pathway (PPP), whose oxidative pathway is a major source of NADPH for the yeast. The acetaldehyde oxidation into acetic acid by *ALD6* leads to NADPH regeneration and compensates for the PPP imbalance (Erasmus et al. 2003; Erasmus and van Vuuren 2009).

We found that the environmental effects on acetic acid production are similar for the hybrids, although the magnitude of these effects was strain specific. As expected, the two low acetic acid mutants Eg8_V2 and Eg8_V3 have a lower acetic acid production than the other strains under the same environmental conditions. Indeed, Eg8_V2 and Eg8_V3 have been obtained by UV mutagenesis from other strains from the Eg8 family, and selected for their low acetic acid production. In the present study, the production of acetic acid of Eg8_V2 and Eg8_V3 never exceeded 0.7 g/L (Ribéreau-Gayon et al. 2006), described as the concentration before organoleptic quality degradation of the wine occurs. Our study emphasizes the importance of strain choice, in addition to environmental conditions. To optimize the wine fermentation, these two parameters should be considered.

Using Eg8_V2, we also studied the influence of environmental conditions on the final concentration of thiols in wines obtained by fermentations of synthetic and natural grape musts. We found that lipid addition (phytosterols + Tween80 or Tween80 alone) leads to an increase in the final concentration of 3MH in both cases (Figs. 2, 3b). Moreover, in natural grape must, we also found that the higher the initial lipid concentration is, the higher the final 4MMP concentration (Fig. 3a). Pinu et al. (2014) previously showed that adding linolenic acid (four times the initial concentration) to a must leads to a doubling of the 4MMP content and a 17% increase in the 3MH content. The increase in the amounts of 3MH and 4MMP released as the lipid concentration is increased could be explained by the antioxidant properties of lipid nutrients (Landolfo et al. 2010). Indeed, it has been demonstrated that adding antioxidant molecules (120 mg/kg SO_2_) to the grape must leads to an increase in final thiol concentrations (Saharan et al. 2010).

Moreover, we observed in this study a decrease in 3MHA production when the lipid concentrations were increased. However, we showed that the global production of 3MH (3MH + 3MHA) was not impacted by the lipid concentration (Fig. 3e). This suggests an increase in the conversion of 3MH into 3MHA when the lipid content is low, leading to a decrease in the 3MH/3MHA ratio. For example, at 24 °C, the 3MH/3MHA ratio drops from 7.03 to 2.29 when the lipid content decreases from 0.0533 to 0.0133% v/v (Fig. 3d). One potential reason is that the conversion of 3MH into 3MHA is catalyzed by an alcohol acetyltransferase encoded by *ATF1* (Verstrepen et al. 2003), whose expression is repressed by unsaturated fatty acids (Fujii et al. 1997). Moreover, 3MHA is the product of acetylation between 3MH and acetyl-CoA (Swiegers and Pretorius 2007). We previously hypothesized that lipids limitation leads to increased production of cytosolic acetyl-CoA, which is required for fatty acid production in anaerobiosis. A greater availability of acetyl-CoA could also explain the higher 3MH acetylation when lipid concentration decreases.

The reduced acetylation of 3MH, along with the high concentrations of lipids observed in our study (Fig. 3d, e), results in the modification of the aromatic profile from one with a passion fruit aroma brought about by the 3MHA to one with a citrus aroma brought by the 3MH, which could be interesting for winemakers.

To conclude, we demonstrated that the lipid content of the must is critical for wine fermentations performed by *S. cerevisiae* × *S. kudriavzevii* hybrid strains from the Eg8 cluster. Indeed, when Eg8 hybrids undergo lipid deprivation, their acetic acid production increases and their liberation of thiols is modified, leading to more passion fruit aromas and less box tree and citrus notes (the 4MMP released is lower and the acetylation of 3MH increases). Therefore, as the clarification of the must is critical for the lipid content (Cocito and Delfini 1997), a must clarification strategy needs to be chosen according to the aromatic profile that the winemaker is searching for while minimizing the risk of excessive acetic acid production. Further improvement of these strains needs to be performed in order to obtain new starters that can maximize 3MHA production without the risk of excessive acetic acid production.

## Additional file

**Additional file 1.** Additional figures and tables.

## Abbreviations

ACDH: acetaldehyde dehydrogenase
Cys: cysteine
EI: electronic impact
GC: gas chromotography
Glu: glutathione
MS: mass spectrometry
NanoLC: nano liquid chromatography
NTU: nephelometric turbidity unit
PDH: pyruvate dehydrogenase
PI: propidium iodide
PPP: pentose phosphate pathway
*S. cerevisiae*: *Saccharomyces cerevisiae*
SIDA: stable isotope dilution assay
*S. kudriavzevii*: *Saccharomyces kudriavzevii*
SM: synthetic must
SNK: Student–Newman and Keuls
SPME: solid-phase microextraction
V_max_: maximum fermentation rate
v/v: volume/volume
w/v: weight/volume
YAN: yeast available nitrogen
YPD: yeast extract peptone dextrose
3MH: 3-mercaptohexan-1-ol
3MHA: 3-mercaptohexyl acetate
4MMP: 4-methyl-4-mercaptopentan-2-one
