# Supplementary material for: Lipids modulate acetic acid and thiol final concentrations in wine during fermentation by Saccharomyces cerevisiae × Saccharomyces kudriavzevii hybrids
Source: AMB Express. 2018 Aug 10;8:130. doi: 10.1186/s13568-018-0657-5 (PMC6086921; doi:10.1186/s13568-018-0657-5)
Supplement: Supplementary file 1 — Additional file 1. Additional figures and tables. [file 13568_2018_657_MOESM1_ESM.pdf]

Additional file 1

Lipids modulate acetic acid and thiol final concentrations in wine during fermentation by *Saccharomyces cerevisiae* X *Saccharomyces kudriavzevii* hybrids

Applied Microbiology and Biotechnology Express

Amandine Deroite, Jean-Luc Legras, Peggy Rigou, Anne Ortiz-Julien and Sylvie Dequin

Affiliation and e-mail address of the corresponding author:
SPO, Univ Montpellier, INRA, Montpellier SupAgro, Montpellier, France
sylvie.dequin@inra.fr

Additional file 1: Table S1 Raw data obtained in the Box-Behnken experimental design for each studied compound**.** LC: lipid content; T: temperature; S: sugar; X: population; ND: not determined

Strain	Conditions			Yeast		Fermentation		Carbon metabolism (g/L)				Thiols (ng/L)		
	LC (% v/v)	T(°C)	S (g/L)	Viability (%)	X (*10^6 C/mL)	Vmax (g/L/h)	End of fermentation (h)	Acetic acid	Succinic acid	Glycerol	Pyruvic acid	4MMP	3MH	3MHA
Eg8_V0	0.0133	16	210	72.8	64.8	0.7	474	0.665	0.180	4.690	0.069			
	0.0533	16	210	95.7	102.5	0.7	320	0.476	0.257	4.770	0.192			
	0.0133	24	210	60.2	70.0	1.3	ND	0.724	0.243	5.481	0.093			
	0.0533	24	210	96.7	75.1	1.3	186	0.563	0.294	5.520	0.106			
	0.0133	20	170	87.3	74.9	1.1	190	0.450	0.211	4.463	0.332			
	0.0533	20	170	95.2	92.1	1.0	164	0.364	0.282	4.922	0.372			
	0.0133	20	250	44.3	70.0	0.9	ND	0.554	0.602	6.640	0.037			
	0.0533	20	250	92.1	89.4	1.0	ND	0.513	0.348	6.352	0.082			
	0.0333	16	170	99.0	92.5	0.7	245	0.383	0.212	4.285	0.070			
	0.0333	24	170	98.6	88.5	1.5	127	0.506	0.253	5.175	0.133			
	0.0333	16	250	91.5	84.7	0.7	ND	0.737	0.246	5.583	0.291			
	0.0333	24	250	49.4	73.6	1.3	ND	0.959	0.312	6.690	0.059			
	0.0333	20	210	97.5	79.8	1.0	219	0.586	0.267	5.401	0.297			
	0.0333	20	210	97.1	80.4	1.0	219	0.559	0.266	5.283	0.435			
	0.0333	20	210	94.2	85.9	1.0	219	0.528	0.279	5.420	0.439			
	0.0333	20	210	97.6	82.9	1.0	219	0.559	0.278	5.422	0.434			
Eg8_V2	0.0133	16	210	86.5	62.5	0.7	362	0.559	0.197	4.876	0.099	194	108	31
	0.0533	16	210	95.2	98.1	0.8	325	0.425	0.268	4.987	0.137	52	251	25
	0.0133	24	210	56.3	76.3	1.3	215	0.524	0.290	5.721	0.146	53	206	34
	0.0533	24	210	93.9	94.4	1.4	141	0.197	0.358	5.854	0.238	88	212	24
	0.0133	20	170	63.1	70.5	1.0	200	0.353	0.195	4.218	0.133	86	106	40
	0.0533	20	170	98.8	112.1	1.1	143	0.240	0.284	4.612	0.175	208	277	33
	0.0133	20	250	52.8	63.3	0.9	ND	0.665	0.276	6.172	0.129	82	126	46
	0.0533	20	250	88.7	92.0	1.0	283	0.517	0.394	6.563	0.175	178	290	32
	0.0333	16	170	92.9	106.2	0.8	220	0.264	0.216	4.135	0.132	179	258	56
	0.0333	24	170	91.3	94.1	1.4	112	0.229	0.257	5.402	0.102	87	209	18
	0.0333	16	250	84.2	78.0	0.7	473	0.634	0.288	4.727	0.162	91	190	46
	0.0333	24	250	66.1	70.8	1.3	235	0.616	0.367	6.698	0.200	59	183	27
	0.0333	20	210	91.8	88.3	1.0	210	0.340	0.304	5.386	0.165	129	154	26
	0.0333	20	210	92.4	86.2	1.1	210	0.378	0.299	5.386	0.183	129	134	22
	0.0333	20	210	90.3	81.0	1.1	210	0.263	0.223	3.994	0.129	130	154	28
	0.0333	20	210	89.4	78.7	1.1	210	0.385	0.305	5.506	0.186			
Eg8_V3	0.0133	16	210	74.3	50.0	0.6	474	0.439	0.190	4.559	0.081			
	0.0533	16	210	97.6	101.6	0.7	323	0.313	0.249	4.745	0.105			
	0.0133	24	210	52.7	68.3	1.3	201	0.434	0.269	5.622	0.155			
	0.0533	24	210	93.2	99.5	1.4	144	0.310	0.357	5.946	0.181			
	0.0133	20	170	74.6	68.2	1.1	200	0.207	0.213	4.154	0.194			
	0.0533	20	170	97.4	114.1	1.1	149	0.190	0.282	4.710	0.296			
	0.0133	20	250	45.2	66.5	1.0	ND	0.567	0.269	6.129	0.175			
	0.0533	20	250	94.5	114.7	1.0	283	0.457	0.375	6.563	0.220			
	0.0333	16	170	93.6	110.5	0.8	235	0.230	0.204	4.117	0.098			
	0.0333	24	170	93.8	137.8	1.5	108	0.228	0.269	4.716	0.323			
	0.0333	16	250	80.9	62.3	0.7	474	0.620	0.179	5.457	0.067			
	0.0333	24	250	79.0	77.0	1.4	200	0.485	0.361	6.459	0.151			
	0.0333	20	210	91.6	85.4	1.0	210	0.313	0.303	5.507	0.345			
	0.0333	20	210	87.0	86.4	1.0	210	0.260	0.297	5.304	0.361			
	0.0333	20	210	87.9	79.5	1.0	210	0.269	0.300	5.412	0.347			
	0.0333	20	210	85.1	84.5	1.0	210	0.313	0.296	5.481	0.383			
Eg8_V4	0.0133	16	210	77.8	65.3	0.7	474	0.681	0.165	4.553	0.060			
	0.0533	16	210	93.1	98.4	0.7	385	0.569	0.210	4.760	0.084			
	0.0133	24	210	50.3	66.0	1.3	236	0.903	0.172	5.026	0.082			
	0.0533	24	210	98.1	97.6	1.4	175	0.634	0.243	5.133	0.167			
	0.0133	20	170	89.1	88.4	1.0	179	0.510	0.153	4.074	0.089			
	0.0533	20	170	98.5	105.5	1.1	162	0.454	0.197	4.321	0.101			
	0.0133	20	250	92.6	94.4	1.1	ND	0.865	0.272	6.320	0.104			
	0.0533	20	250	98.5	96.7	1.1	ND	0.861	0.155	6.108	0.076			
	0.0333	16	170	95.7	103.1	0.8	258	0.451	0.178	4.063	0.086			
	0.0333	24	170	97.1	112.8	1.4	124	0.490	0.182	4.170	0.136			
	0.0333	16	250	94.5	89.9	0.7	474	0.752	0.209	5.264	0.048			
	0.0333	24	250	74.1	86.8	1.4	160	0.907	0.257	5.949	0.175			
	0.0333	20	210	95.4	102.5	1.0	251	0.628	0.234	5.086	0.077			
	0.0333	20	210	98.0	87.7	1.0	251	0.603	0.211	5.157	0.082			
	0.0333	20	210	96.7	86.9	1.1	251	0.607	0.211	5.273	0.108			
	0.0333	20	210	96.2	88.4	1.0	251	0.596	0.214	5.129	0.138			

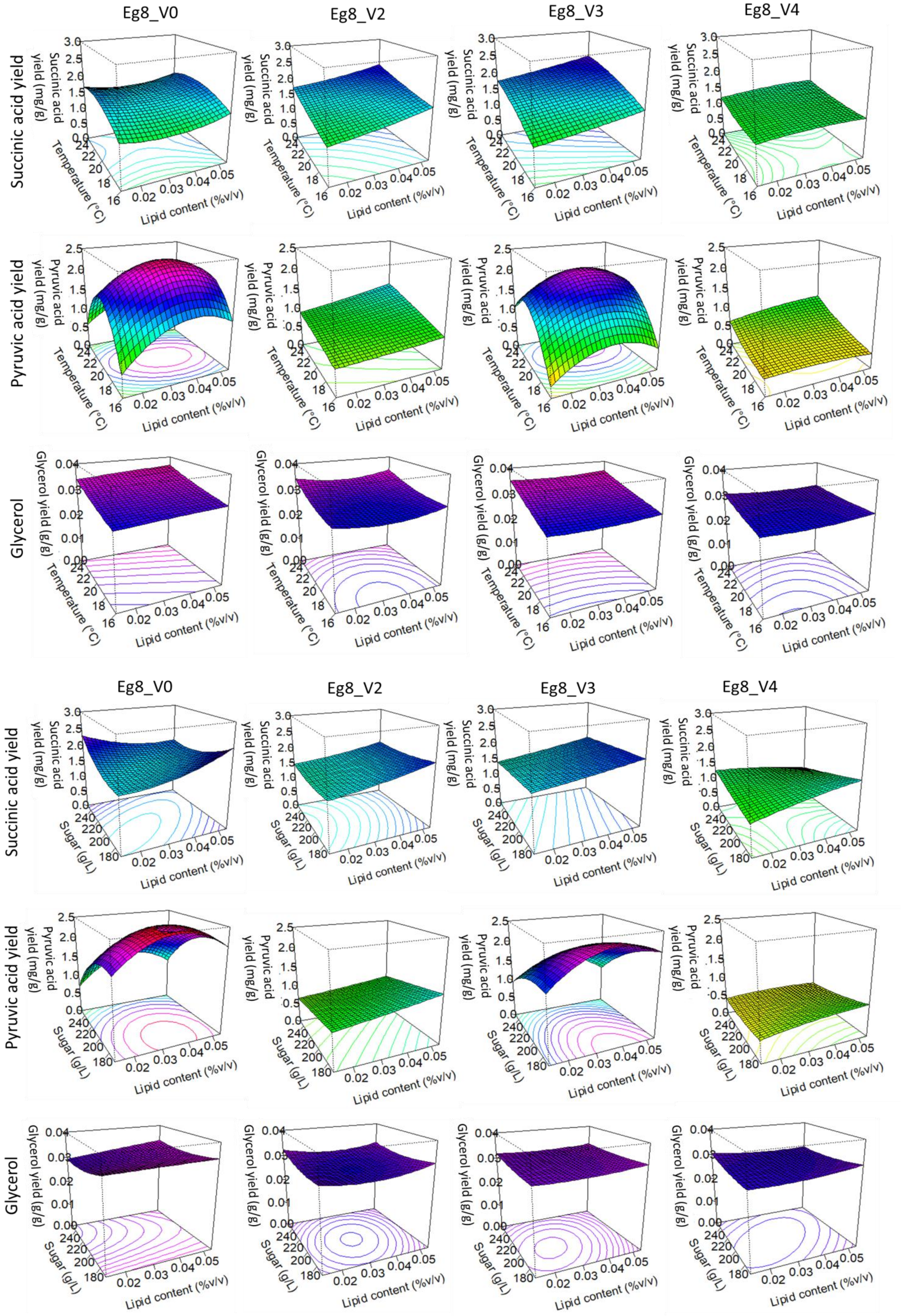

Additional file 1: Fig. S1: Evolution of the yields of succinic acid (mg/g), pyruvic acid (mg/g) and glycerol (g/g) of *S. cerevisiae* X *S. kudriavzevii* hybrids Eg8_V0, Eg8_V2, Eg8_V3 and Eg8_V4 under different environmental conditions

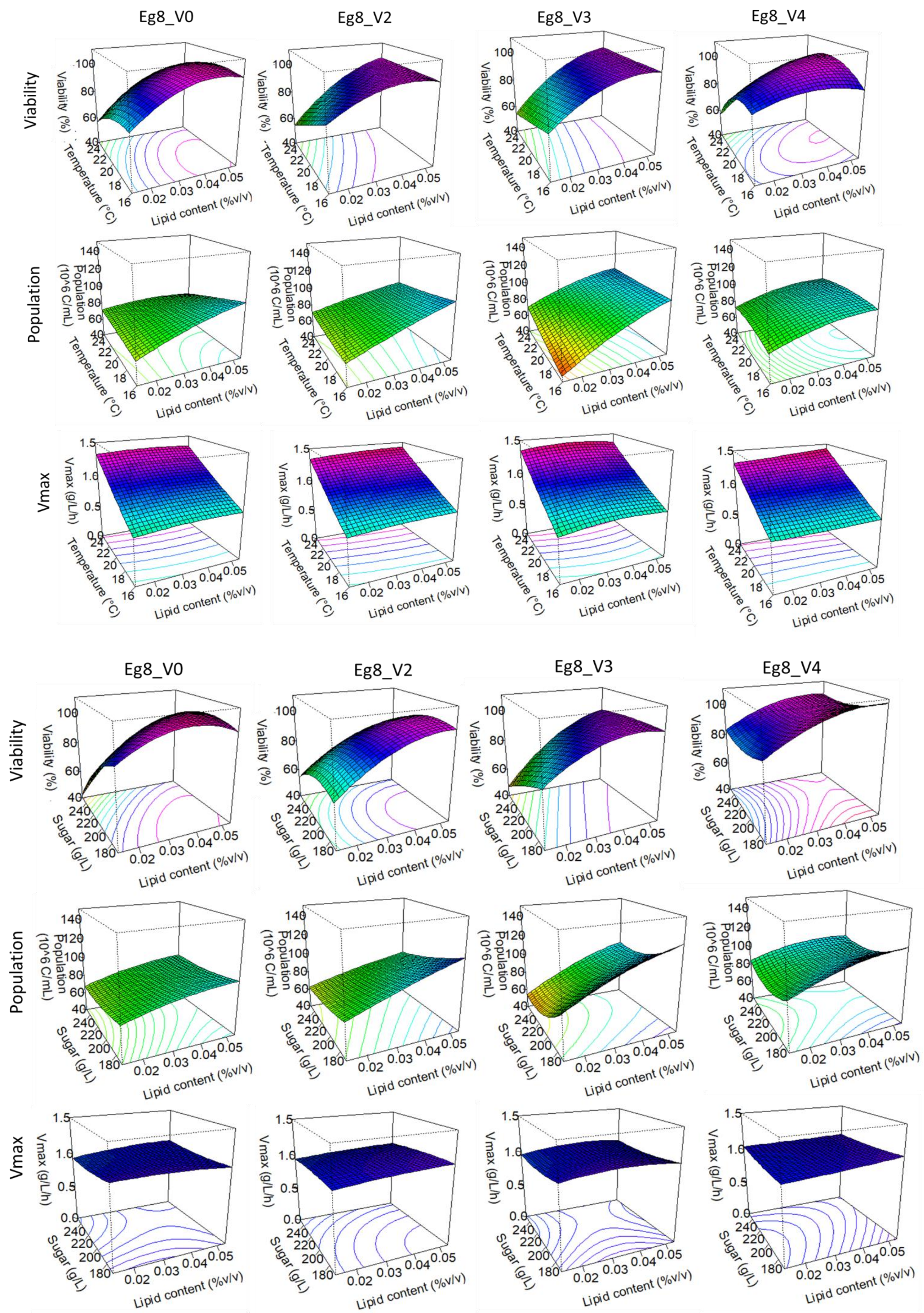

Additional file 1: Fig. S2: Evolution of viability (%), population (*10^6 C/mL) and V_{max} (gCO_2/L.h) of *S. cerevisiae* X *S. kudriavzevii* hybrids Eg8_V0, Eg8_V2, Eg8_V3 and Eg8_V4 under different environmental conditions

Additional file 1: Table S2 Raw data obtained in the confirmation in Sauvignon Blanc du Gers 2017 before clarification and chaptalization for each studied compound measured when 80% of sugar is consumed (Phyto=phytosterols; Temp=temperature)

Lipid content (%v/v)	Phyto	Temp (°C)	Glucose (g/L)	Fructose (g/L)	Acetic acid (g/L)	Succinic acid (g/L)	Pyruvic acid (g/L)	Glycerol (g/L)	4MMP (ng/L)	3MH (ng/L)	3MHA (ng/L)
0.0133	Yes	16	8.93	37.86	0.11	0.58	0.63	6.04	11.87	3273.4	2324.5
			10.55	37.36	0.09	0.51	0.54	5.13	11.00	2890.4	2108.5
			9.95	36.82	0.1	0.51	0.57	5.15	12.97	3317.2	2483.5
0.0333			9.39	35.9	0.11	0.54	0.60	5.32	10.20	3247.8	2160.4
			10.01	36.59	0.1	0.53	0.66	5.24	13.09	3421.3	2184.3
			8.14	34.06	0.11	0.53	0.58	5.32	12.06	3498.9	2260.8
0.0533			9.87	37.1	0.15	0.56	0.65	5.35	10.46	3040.1	1725.2
			10.17	37.17	0.16	0.57	0.61	5.38	10.09	2970.8	1702.2
			9.92	37.09	0.15	0.55	0.61	5.27	12.23	3444.8	2010.2
0.0133		20	5.7	29.12	0.1	0.50	0.61	5.45	16.08	3507.0	1828.9
			6.65	31.07	0.1	0.52	0.58	5.54	14.82	3505.6	1731.2
			7.42	32.37	0.1	0.50	0.65	5.43	16.41	3801.2	1792.9
0.0333			6.17	30.09	0.11	0.54	0.58	5.52	14.95	4125.7	1641.1
			5.08	27.67	0.11	0.53	0.60	5.50	16.34	3833.2	1636.8
			8.41	34.23	0.11	0.52	0.61	5.36	16.96	3450.0	1479.9
0.0533			7.64	32.9	0.14	0.56	0.56	5.45	15.52	3789.0	1259.2
			9.33	35.75	0.15	0.56	0.62	5.40	12.12	3522.3	1179.4
			9.48	35.98	0.14	0.56	0.65	5.36	14.96	3930.2	1277.6
0.0133		24	8.89	34.85	0.1	0.51	0.58	5.56	19.61	4586.4	1261.7
			8.26	41.35	0.14	0.72	0.81	8.06	20.02	4570.7	1208.4
			6.18	29.35	0.1	0.50	0.56	5.64	19.72	4625.4	1393.7
0.0333			6.89	31.15	0.12	0.55	0.61	5.69	17.62	4010.0	962.6
			9.6	35.6	0.12	0.53	0.62	5.46	17.72	3826.4	858.9
			8.89	34.52	0.12	0.53	0.61	5.57	17.53	3966.6	877.6
0.0533			9.64	35.72	0.16	0.57	0.65	5.54	17.05	4221.6	761.3
			5.96	26.62	0.14	0.49	0.61	4.81	16.02	4188.1	865.0
			5.11	27.66	0.17	0.59	0.61	5.84	17.70	4896.1	862.2
0.0133	No	20	7.04	31.5	0.11	0.49	0.68	5.48	12.44	4047.9	2448.7
			7.13	31.76	0.1	0.49	0.58	5.47	12.17	3877.7	2284.1
			4.11	24.72	0.1	0.49	0.58	5.35	10.85	3796.6	2227.9
0.0333			10.63	37.74	0.13	0.53	0.663	5.37	18.18	4481.1	1544.7
			6.24	30.22	0.13	0.54	0.59	5.46	15.73	4101.4	1710.2
			7.87	33.5	0.13	0.53	0.63	5.40	15.46	4546.9	1672.3
0.0533			6.53	30.79	0.16	0.54	0.62	5.42	19.39	4691.9	1436.7
			5.13	27.99	0.18	0.56	0.63	5.57	18.30	5102.9	1334.7
			7.89	33.39	0.17	0.55	0.61	5.32	17.89	5300.7	1263.1

Additional file 1: Table S3 Raw data obtained for the confirmation in Sauvignon Blanc du Gers 2017 after clarification to 20 NTU and chaptalization to 240 g/L sugar at 20°C for each studied compound measured when 80% of sugar is consumed

Lipid content (%v/v)	Phytosterols	Glucose (g/L)	Fructose (g/L)	Acetic acid (g/L)	Succinic acid (g/L)	Pyruvic acid(g/L)	Glycerol (g/L)
0.0133	Yes	8.21	40.50	0.28	0.58	0.44	7.53
		5.62	34.73	0.35	0.53	0.41	7.48
		7.90	40.19	0.27	0.58	0.40	7.64
0.0333		10.19	44.37	0.20	0.63	0.47	7.41
		7.68	39.82	0.21	0.66	0.47	7.81
		10.58	45.34	0.19	0.66	0.46	7.76
0.0533		8.27	41.10	0.18	0.73	0.44	8.06
		8.52	41.73	0.18	0.69	0.47	7.70
		8.50	41.67	0.17	0.70	0.46	7.73
0.0133	No	9.98	44.23	0.32	0.57	0.40	7.75
		9.75	43.82	0.32	0.57	0.38	7.67
		8.11	40.59	0.40	0.51	0.41	7.36
0.0333		16.82	55.21	0.20	0.68	0.41	8.09
		15.06	52.64	0.18	0.73	0.41	8.00
		16.29	54.23	0.24	0.63	0.41	7.60
0.0533		9.98	44.22	0.33	0.59	0.44	7.41
		12.36	48.57	0.21	0.71	0.43	8.17
		14.56	52.09	0.28	0.68	0.42	7.89
